# Patterns and Clinical Outcomes of Sitagliptin/Metformin Extended-Release in Internal Medicine: A Real-World Multicenter Italian Study

**DOI:** 10.3390/jcm15030927

**Published:** 2026-01-23

**Authors:** Mariarosaria De Luca, Michele Arcopinto, Giosiana Bosco, Sebastiano Cicco, Francesco Di Giacomo Barbagallo, Chiara Giacinti, Marialuisa Sveva Marozzi, Maristella Salvatora Masala, Miriam Pinna, Giacomo Pucci, Andrea Salzano, Roberto Scicali, Alberto Maria Marra, Antonio Cittadini

**Affiliations:** 1 Department of Translational Medical Sciences, University of Naples “Federico II”, Via Pansini n.5, 80127 Naples, Italy; mariarosaria.deluca@unina.it (M.D.L.); michele.arcopinto83@unina.it (M.A.); andrea.salzano@unina.it (A.S.); antonio.cittadini@unina.it (A.C.); 2 Department of Clinical and Experimental Medicine, University of Catania, 95123 Catania, Italy; giosiana.bosco@gmail.com (G.B.); fdigiacomobarbagallo@gmail.com (F.D.G.B.); robertoscicali@gmail.com (R.S.); 3 Department of Medicine and Surgery, “Kore” University of Enna, 94100 Enna, Italy; 4 Unit of Internal Medicine “Guido Baccelli” and Arterial Hypertension “Anna Maria Pirrelli”, Department of Precision and Regenerative Medicine and Ionian Area (DiMePRe-J), University of Bari Aldo Moro, University Hospital Policlinico Di Bari, 70124 Bari, Italy; sebastiano.cicco@uniba.it (S.C.); marialuisa.marozzi@gmail.com (M.S.M.); 5 Department of Medicine and Surgery, University of Perugia, 06123 Terni, Italy; chiaragiacinti88@gmail.com (C.G.); giacomo.pucci@unipg.it (G.P.); 6 Metabolic Diseases and Diabetology, Health District of Sassari, 07100 Sassari, Italy; maristella.masala@yahoo.com (M.S.M.); miriam.pinna@aslsassari.it (M.P.)

**Keywords:** type 2 diabetes mellitus, sitagliptin/metformin, fixed-dose combination, real-world evidence, internal medicine

## Abstract

**Background:** In internal medicine, the management of type 2 diabetes mellitus (T2DM) is challenged by multimorbidity and polypharmacy. The fixed-dose combination of sitagliptin and extended-release metformin (SITA/MET ER) is a valuable option for frail and comorbid patients. **Methods:** This multicenter, retrospective, observational study involved five Italian Internal Medicine units. Consecutive patients with T2DM who initiated SITA/MET ER were included. Demographic, clinical, and laboratory data were collected at baseline (T0) and at follow-up (T1, 3–4 months). The primary endpoint was change in HbA1c; secondary endpoints included fasting plasma glucose (FPG), treatment adherence, adverse events, and modifications in concomitant antidiabetic therapies. **Results:** A total of 292 patients (mean age 70.8 ± 10.6 years; 43% female) were analyzed. At baseline, mean HbA1c was 7.4 ± 1.0% and FPG 150.2 ± 42.5 mg/dL, with significant reductions observed at follow-up (HbA1c 7.0 ± 0.8%, FPG 136.8 ± 29.6 mg/dL; both *p* < 0.05). SITA/MET ER was predominantly prescribed to patients with a complex clinical profile, as reflected by the high prevalence of microvascular (37%) and macrovascular (42%) complications. The use of sulfonylureas decreased from 11% to 3% (*p* < 0.001), while SGLT2 inhibitor and insulin use remained stable. Treatment adherence to SITA/MET ER was excellent, with full compliance reported and no adverse events recorded. **Conclusions:** In this real-world internal medicine study, SITA/MET ER improved glycemic control and was well tolerated among patients with complex clinical profiles. These findings support the role of SITA/MET ER as a flexible and practical therapeutic choice in this setting.

## 1. Introduction

The prevalence of type 2 diabetes mellitus (T2DM) is steadily increasing worldwide with a significant proportion of patients managed in internal medicine setting (wards and outpatient clinics) [1]. Internists frequently encounter patients with more complex clinical profiles, characterized by multiple comorbidities, polypharmacy, and varying degrees of frailty [2]. Therefore, despite the availability of newer antidiabetic agents, therapeutic decisions often prioritize safety, tolerability, and treatment simplification over strict glycemic targets, in line with current recommendations for complex and elderly patients [3]. In this context, Sitagliptin combined with extended-release metformin (SITA/MET ER) represents a therapeutic option within the armamentarium of antidiabetic treatments that may be particularly suitable for complex patients [4]. DPP-4 inhibitors are associated with a low risk of hypoglycemia, neutral effects on body weight, and a favorable safety profile in elderly and multimorbid patients, while the extended-release formulation of metformin improves gastrointestinal tolerability and treatment adherence compared with the immediate-release formulations. This combination is especially relevant for patients with high or very high cardiovascular risk who are intolerant to GLP-1 receptor agonists, intolerant to immediate-release metformin [5], already receiving SGLT2 inhibitors but requiring improved glycemic control, patients with poor adherence to complex combination therapies [6], and those treated with sulfonylureas, as a potential alternative to facilitate sulfonylurea withdrawal [7].

Although several randomized clinical trials and real-world studies have evaluated the efficacy and safety of DPP-4 inhibitors and combinations of sitagliptin and metformin, most available evidence derives from endocrinology or diabetology outpatient settings. However, patients managed in Internal Medicine departments represent a distinct and often underrepresented population [8]. In this setting, therapeutic priorities frequently differ from those of specialized diabetes clinics. Consequently, data generated in diabetology outpatient cohorts may not be fully generalizable to the Internal Medicine population. To date, real-world evidence specifically addressing the use of fixed-dose sitagliptin/metformin extended-release combinations in Internal Medicine settings remains limited.

The aim of the present multicenter Italian observational study was to explore the real-world effectiveness and safety of the fixed-dose combination of sitagliptin/metformin extended-release in internal medicine settings, as well as to describe clinicians’ prescribing patterns. Specifically, the study assessed changes in glycemic control, treatment adherence, tolerability, adverse events, and modifications of concomitant antidiabetic therapies, while characterizing the clinical profiles of patients receiving this therapy in routine practice.

## 2. Materials and Methods

This was a multicenter, retrospective, observational study conducted in five Internal Medicine units across Italy (Appendix A). The real-world prescribing patterns for the fixed-dose combination of SITA/MET ER, regardless of the prescribed dose, were recorded, along with its effectiveness, adherence, and safety in patients with type 2 diabetes mellitus (T2DM) managed in internal medicine settings.

All consecutive adult patients aged 18 years or older who initiated therapy with SITA/MET ER between January and June 2025 were included. No specific exclusion criteria were applied, in order to capture a broad and unselected real-world population. Patients had been managed during hospitalization, day-hospital stay, or outpatient visits in Internal Medicine departments, and had a follow-up visit within 4 months from treatment initiation. No predefined treatment algorithms were applied, and therapeutic decisions were entirely at the discretion of the treating physicians, according to local practice and current clinical judgment.

For each patient, demographic, clinical, laboratory, and therapeutic data were collected at baseline (T0, before initiating therapy) and at follow-up (T1, approximately 3–4 months (90–120 days). Time points (T0 and T1) were defined retrospectively for analytical purposes, based on the timing of visits documented in medical records, rather than a predefined follow-up schedule. For patients whose follow-up visit fell outside this interval, data were included if the visit occurred within a maximum window of 150 days from baseline. Although follow-up visits could occur up to 150 days from baseline, HbA1c measurements used for the analysis were those performed at approximately 3 months after treatment initiation, in line with routine laboratory monitoring. When multiple measurements were available, the value closest to 90 days was selected. Patients with follow-up visits beyond this period were excluded from the analysis of glycemic outcomes to maintain consistency in the assessment interval. Data included general patient characteristics, diabetes-related parameters, microvascular comorbidities (including retinopathy, nephropathy, and neuropathy), macrovascular comorbidities (including coronary artery disease, cerebrovascular disease, and peripheral artery disease), ongoing medications, and treatment adherence. Renal function was assessed using the CKD-EPI equation. Changes in therapy were recorded to characterize real-world prescribing practices. Adherence was assessed based on retrospective chart review and documented patient reports. Full adherence was defined as continuous use of SITA/MET ER without reported interruptions or dose reductions between T0 and T1.

The primary outcome was the change in HbA1c from baseline to follow-up. Secondary outcomes included fasting plasma glucose (FPG), treatment adherence, adverse events, and modifications in concomitant antidiabetic therapies. Adverse events included: symptomatic or laboratory-confirmed hypoglycemia, gastrointestinal events that required specific therapy, medical consultation, or hospitalization (such as nausea, vomiting, diarrhea, or abdominal discomfort), and cardiovascular events, including acute coronary syndrome, heart failure exacerbation, arrhythmias, or cerebrovascular events. All events were identified through review of clinical records, patient reports, and laboratory results. Given the retrospective nature of the study, mild or non-clinically reported adverse events may not have been captured, as is common in real-world practice.

### 2.1. Statistical Analysis

Continuous variables were expressed as mean ± standard deviation (SD) or as median (interquartile range), depending on their distribution. Comparisons between baseline and follow-up values were performed using the paired Student’s *t*-test for normally distributed variables and the Wilcoxon signed-rank test for non-normally distributed variables. Categorical variables were expressed as absolute numbers and percentages and compared using the chi-square test or Fisher’s exact test, as appropriate. A *p*-value < 0.05 was considered statistically significant. Statistical analysis was performed using R version 3.0 (http://www.r-project.org).

### 2.2. Ethical Considerations

The study protocol was approved by the Ethics Committees of Federico II University (Protocol No. 63/2024, approval date: 19 November 2025) and was conducted in accordance with the principles of the Declaration of Helsinki. Given its retrospective observational nature, formal informed consents were waived according to national regulations, provided that patient data were anonymized prior to analysis.

## 3. Results

A total of 292 patients were included in the study. At baseline (T0), 31 out of 292 patients were managed in an Internal Medicine outpatient setting, whereas the remaining patients were evaluated during ordinary hospitalization or day-hospital admission in Internal Medicine units. All patients had a documented outpatient follow-up visit in the outpatient setting at follow-up (T1), corresponding to a post-discharge visit performed 3–4 months after initiation of SITA/MET ER therapy.

The mean age of the cohort was 70.8 ± 10.6 years, and 105 patients (36%) were aged over 75 years. Forty-three percent of the cohort were female. The median duration of diabetes was 12.2 ± 9.4 years. Patients presented with a wide range of comorbidities (37% macrovascular complications, 42% microvascular complications). No clinically meaningful changes in body mass index were observed, with BMI values of 25.9 ± 4.8 kg/m^2^ at baseline and 25.8 ± 4.6 kg/m^2^ at follow-up.

At baseline (T0), mean HbA1c was 7.4 ± 1.0%, and mean fasting plasma glucose was 150.2 ± 42.5 mg/dL. Significant improvements in glycemic control were observed at follow-up (T1).

**Figure 1:**
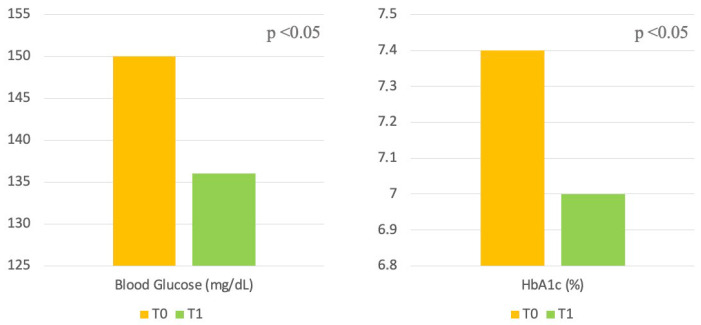
Changes in blood glucose and HbA1c from Baseline (T0) to End of Study (T1).

Mean HbA1c decreased to 7.0 ± 0.8% (*p* < 0.05) and mean fasting plasma glucose decreased to 136.8 ± 29.6 mg/dL (*p* < 0.05) (Table 1 and Figure 1). No significant changes in renal function, as assessed by eGFR, were observed during the follow-up period.

**Figure 2:**
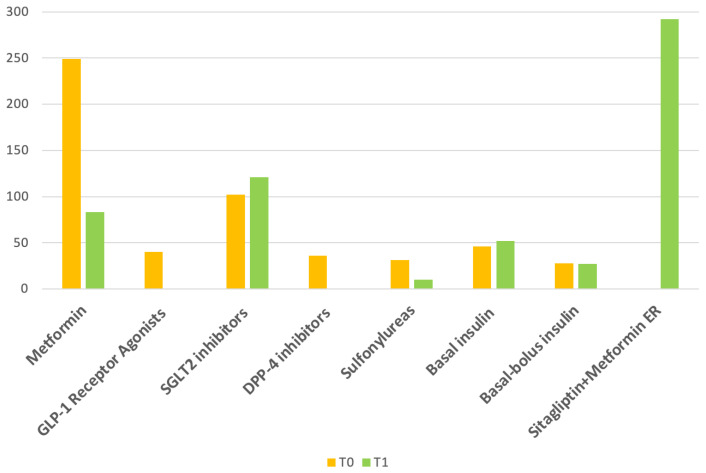
Changes in anti-diabetic therapies from Baseline (T0) to Follow-up visit (T1).

At T0 and T1, the use of SGLT2 inhibitors was similar between the two time points (35% vs. 41.4%, *p* = ns). At baseline, 40 out of 292 patients (13.7%) were receiving GLP-1 receptor agonists, while none were on treatment at follow-up. As expected, the use of DPP-4 inhibitors dropped to 0% at follow-up, since all patients had been started on the fixed-dose combination of sitagliptin and metformin, and therefore the addition of a second DPP-4 inhibitor was not recommended. The use of sulfonylureas declined from 11% to 3% (*p* < 0.001). No significant changes were observed in the use of basal insulin (15% vs. 18%, *p* = ns) or basal-bolus insulin (10% vs. 9%, *p* = ns) between T0 and T1 (Table 1, Figure 2).

Adherence was considered high, as no interruptions or dose changes were reported in medical charts. No clinically documented adverse events were identified among patients with available follow-up data within the predefined timeframe.

## 4. Discussion

This multicenter, real-world, Italian observational study conducted in internal medicine settings provides novel insights into the clinical patterns of patients treated with the fixed-dose combination of SITA/MET ER, as well as its effectiveness, safety, and tolerability in routine practice. The main findings of the present investigation confirm indeed that this therapeutic option is effective in improving glycemic control and is well tolerated among a population characterized by clinical complexity and multiple comorbidities [4].

Significant improvements in HbA1c and fasting plasma glucose were observed at follow-up. Although other adjustments in concomitant therapies, including reductions in sulfonylureas and modest changes in insulin regimens, also occurred, the initiation of SITA/MET ER represented the most substantial therapeutic change and is therefore likely to have contributed predominantly to the observed improvements in glycemic control. Although the absolute reduction in HbA1c was modest, it was achieved in a population with long-standing diabetes and high comorbidity burden. Importantly, the possibility of reducing or even discontinuing sulfonylureas and lowering insulin requirements following SITA/MET ER initiation represents an additional clinical advantage, allowing for simplification of therapy and a lower risk of hypoglycemia, which is particularly relevant in elderly and multimorbid patients [9,10]. These results are particularly relevant for internal medicine settings, where patients are often older, frail, and have multiple comorbidities, a population that is underrepresented in traditional outpatient diabetes trials. Real-world evidence from this setting provides insights that cannot be extrapolated from specialized diabetes clinics.

Because type 2 diabetes is a progressive disease, combination therapy is often essential to achieve and maintain glycemic targets, and current international guidelines increasingly recommend early or simultaneous use of two or more agents to address multiple pathophysiological defects and improve attainment of individualized treatment goals [11]. This approach allows for more effective and durable glycemic control while minimizing the need for therapies associated with hypoglycemia or weight gain, particularly in patients with high cardiovascular or renal risk. The results of the present study support the role of the combination as a practical and effective strategy for optimizing glycemia in patients who are often difficult to manage due to polypharmacy, multiple comorbidities, and intolerance to other antidiabetic agents, and are consistent with previous studies demonstrating the efficacy of DPP-4 inhibitors in combination with metformin for achieving and maintaining glycemic targets when lifestyle measures and prior therapies have been insufficient [4,12].

The study also provides important insights into real-world prescribing patterns in internal medicine, providing the phenotype of the patients who SITA/MET ER was preferentially initiated, i.e., patients with multiple comorbidities, not at glycemic target [13]. Moreover, clinicians considered SITA/MET ER a strategy to replace sulfonylureas, as demonstrated by the marked reduction in the number of patients receiving sulfonylureas at the follow-up visit. This therapeutic shift further underlines the favorable profile of SITA/MET ER in clinical practice, offering a safer and more tolerable alternative to agents associated with a higher risk of hypoglycemia [14]. It was also commonly used as an add-on to basal or basal-bolus insulin, allowing for reductions in bolus doses or total insulin requirements, and in patients already receiving SGLT2 inhibitors who required additional glycemic control, serving also as a potential adjunct to mitigate genitourinary side effects [15,16,17]. Another notable observation is that at baseline only a small proportion of patients (40 out of 292) were receiving GLP-1 receptor agonists, which were subsequently discontinued and replaced with sitagliptin in combination therapy. Given that GLP-1 receptor agonists are considered a first-line option for the treatment of type 2 diabetes in many patients, this low percentage suggests that the vast majority were not candidates for these agents, likely due to prior intolerance, contraindications, or specific clinical considerations such as low body weight, frailty, or other factors limiting their use [11]. SITA/MET ER therefore appears to offer a flexible and well-tolerated alternative in patients for whom GLP-1 receptor agonists are unsuitable [18]. However, reasons for GLP-1 RA discontinuation were not systematically recorded.

These patterns suggest that internists select SITA/MET ER in patients with complex clinical profiles where efficacy, safety, and adherence must be carefully balanced.

The absence of reported adverse events aligns with the known tolerability of both agents and supports the suitability of this regimen in elderly or frail patients, who represent a substantial proportion of the internal medicine population [19,20]. It should be noted, however, that the absence of documented adverse events in this study refers only to patients with follow-up data within the predefined timeframe. Patients who discontinued therapy earlier or were lost to follow-up may not have been captured, which could lead to underestimation of adverse events. Nevertheless, the observed safety profile is consistent with the well-established tolerability of sitagliptin and metformin ER, supporting its suitability for use in complex internal medicine patients. The low risk of hypoglycemia and weight neutrality associated with DPP-4 inhibitors further enhance their appropriateness in multimorbid individuals. However, being a retrospective study, mild or non-clinically reported events may have been under-reported, which is a common limitation in real-world practice.

Overall, these findings reinforce the role of SITA/MET ER as a flexible, well-tolerated, and effective option for patients with complex clinical profiles and for whom other therapies may be limited by efficacy, safety, or adherence concerns, particularly typical of the internal medicine setting.

### Limitations

This study has several limitations related to its retrospective and observational design, including the absence of a control group and the relatively short follow-up period. Data were obtained from routine clinical records, which may have led to incomplete capture of relevant clinical variables. In particular, information on body weight changes, mild or non-clinically reported hypoglycemic events, and patient-reported outcomes such as quality of life or treatment satisfaction was not systematically available. While HbA1c measurements were generally obtained around 3 months after treatment initiation, variability in the timing of follow-up visits reflects real-world practice and may represent a minor source of heterogeneity. Moreover, lifestyle-related variables, including dietary habits and physical activity, were not consistently recorded and could have influenced glycemic outcomes. Patients with follow-up visits beyond the predefined timeframe were excluded from the analysis of glycemic outcomes. This may have introduced a selection bias, as patients who discontinued SITA/MET ER earlier due to adverse events or other clinical reasons may not have been captured. Consequently, the absence of reported adverse events should be interpreted with caution and cannot be considered evidence of the absence of treatment-related adverse effects. Nevertheless, the multicenter design and the inclusion of a representative real-world internal medicine population support the external validity and generalizability of the findings.

## 5. Conclusions

The fixed-dose combination of sitagliptin and metformin extended-release represents an effective, safe, and well-tolerated therapeutic option for patients with type 2 diabetes Its use may be particularly advantageous in individuals with complex clinical profiles, high comorbidity burden, or intolerance to other glucose-lowering drugs, the typical patient managed in the internal medicine setting. Future prospective studies with longer follow-up are needed to confirm these findings and to better define the role of SITA/MET ER in therapeutic simplification strategies in complex patients.

## Figures and Tables

**Table 1:** Clinical and therapeutic characteristics of the study population (*n* = 292).

	T0	T1	*p*
Age (years)	70.8 ± 10.6	-	-
Female (*n*, %)	126 (43%)	-	-
BMI (kg/m^2^)	25.9 ± 4.8	25.8 ± 4.6	ns
Diabetes duration (years)	12.2 ± 9.4	-	-
Macrovascular complications (*n*, %)	107 (37%)	-	-
Microvascular complications (*n*, %)	123 (42%)	-	-
Blood glucose (mg/dL)	150.2 ± 42.5	136.8 ± 29.6	<0.05
HbA1c (%)	7.4 ± 1.0	7.0 ± 0.8	<0.05
eGFR (mL/min)	75 ± 20	78 ± 20	ns
Metformin (*n*, %)	249 (85%)	83 (29%)	<0.001
GLP-1 Receptor Agonists (*n*, %)	40 (17%)	0 (0%)	
SGLT2 inhibitors (*n*, %)	102 (35%)	121 (41.4%)	ns
DPP-4 inhibitors (*n*, %) ^1^	36 (12%)	0 (0%)	<0.001
Sulfonylureas (*n*, %)	31 (11%)	10 (3%)	<0.001
Basal insulin (*n*, %)	46 (15%)	52 (18%)	ns
Basal-bolus insulin (*n*, %)	28 (10%)	27 (9%)	ns
Sitagliptin + Metformin ER (*n*, %)	0 (0%)	292 (100%)	<0.001

^1^ Excluding the sitagliptin/metformin LA formulation.

## Data Availability

Data may be available from the corresponding author upon reasonable request and with approval from the relevant ethics committees.

## Abbreviations

The following abbreviations are used in this manuscript:

T2DM	Type 2 diabetes mellitus
SITA/MET ER	Sitagliptin/metformin extended-release
HbA1c	Glycated hemoglobin
FPG	Fasting plasma glucose
eGFR	Estimated glomerular filtration rate
GLP-1 RA	Glucagon-like peptide-1 receptor agonist
SGLT2	Sodium–glucose cotransporter 2
DPP-4	Dipeptidyl peptidase-4
SD	Standard deviation
T0	Baseline
T1	Follow-up

## Appendix A

CLUB Study-group: Marta Apicella ^1^, Antonella Conte ^1^, Francesco Corvasce ^4^, Roberta D’Assante ^1^, Luisa De Rosa ^1^, Dino Della Ventura ^1^, Caterina Divella ^4^, Roberta Frate ^1^, Francesca Giacon ^1^, Silvia Giombini ^4^, Fabrizio Grillo ^1^, Giordana Gaia Iannibelli ^1^, Salvatore Nerino ^1^, Francesca Nocera ^1^, Simona Persia ^4^, Manlio Spagnuolo ^1^, Assunta Stragapede ^4^.
